# Temporal patterns in severe hemoptysis requiring bronchial artery embolization

**DOI:** 10.1186/2049-6958-7-50

**Published:** 2012-12-05

**Authors:** Ignasi Garcia-Olivé, Jose Antonio Fiz, Jose Sanz-Santos, Felipe Andreo, Estefanía Sánchez-Martínez, Jaume Sampere, Jordi Muchart, Josep Maria Michavila, Juan Ruiz-Manzano

**Affiliations:** 1 Respiratory Service, Hospital Universitari Germans Trias i Pujol, Carretera del Canyetsn, Badalona, Barcelona, 08916, Spain; 2 CibeRes – Ciber de Enfermedades Respiratorias, Carretera Soller Km 12, Bunyola, Mallorca, 07110, Spain; 3 Fundació Institut d’Investigació en Ciències de la Salut Germans Trias i Pujol, Carretera del Canyet sn, Badalona, Barcelona, 08916, Spain; 4 Department of Interventional Radiology, Hospital Universitari Germans Trias i Pujol, Carretera del Canyet sn, Badalona, Barcelona, 08916, Spain; 5 Departament de Medicina, Universitat Autònoma de Barcelona, Bellaterra, Barcelona, Spain

**Keywords:** Bronchial artery embolization, Hemoptysis, Seasonal variation, Temporal pattern

## Abstract

**Background:**

Although some authors have suggested that there is some seasonal periodicity in hemoptysis, temporal patterns of hemoptysis have been poorly investigated. The aim of this study is to describe the temporal pattern of severe hemoptysis which required bronchial artery embolization (BAE).

**Methods:**

All consecutive patients with at least one episode of hemoptysis which required BAE during a 13-year period were included. Recurring hemoptysis requiring BAE in a patient with previous embolization was included as a new hemoptysis event, unless it occurred within one month from the prior event. Lineal regression was applied to compute the tendency of occurrence of cases along 13 years of record data. The daily and monthly distributions of embolizations were used to study the weekly and monthly seasonal indexes.

**Results:**

Hemoptysis requiring BAE occurred with some monthly variation demonstrated with two monthly peaks, with the first one occurring during April and the second one during November.

**Conclusion:**

Hemoptysis occurred with two monthly peaks. This seasonal trend might be due to different prevalence of respiratory tract infections or to some weather variables. Identification of significant environmental factors could be useful to improve preventive measures.

## Background

Hemoptysis is a potentially life-threatening occurrence which requires prompt intervention. Several conditions, such as tuberculosis, cancer or bronchiectasis may lead to clinically significant hemoptysis. Although some authors have suggested that there is some seasonal periodicity of hemoptysis [1,2], or association with respiratory tract infections [3-5], temporal patterns of life threatening hemoptysis have been poorly investigated.

Bronchial artery embolization (BAE) is a safe and effective treatment for massive hemoptysis, as well as for chronic but non massive hemoptysis that impairs a patient’s quality of life or can be a precursor of a massive hemoptysis [6,7].

This paper describes the temporal pattern of severe hemoptysis which required bronchial artery embolization.

## Methods

### Patients population

This is an observational retrospective study of patients presenting with life-threatening hemoptysis who underwent BAE at Hospital Universitari Germans Trias i Pujol (HGTiP), a 600-bed tertiary referral hospital in Barcelona (Spain). It is a referral hospital for over 700.000 people. In 2009 there were 27,000 hospital admissions and 110,000 admissions to the emergency room.

All consecutive patients with at least one episode of hemoptysis which required BAE during a 13-year (January 1999-December 2011) period were included. Recurring hemoptysis requiring BAE in a patient with previous embolization was included as a new event, unless it occurred within one month from the previous one (this was considered an early recurrent bleeding) [8].

The research protocol was approved by the regional ethics committee (Ethics Committee for Clinical Research of the Hospital Germans Trias i Pujol).

### Indications for treatment

All patients with massive hemoptysis (i.e a life-threatening hemoptysis) [9,10] were referred to the Interventional Radiology Department for BAE.

### Bronchial arterial embolization

All procedures were performed by two experienced interventional radiologists. A flush descending thoracic aortogram was acquired at the beginning of the procedure followed by selective catheterisation of bronchial arteries using a variety of shaped 5-Fr angiographic catheters. If no abnormal bronchial arteries were identified, non-bronchial systemic arteries were selectively catheterized, depending on the known site of pulmonary disease (assessed by either X-ray, CT-scan or bronchoscopy). Target vessels were super-selectively catheterized using a 3-Fr coaxial microcatheter system if stable cannulation could not be achieved or if more distal cannulation of the vessel was required in order to avoid important side branches, such as the anterior spinal artery. The embolic material used was non-spherical polyvinyl alcohol (PVA) particles or gelatine cross-linked acryl microspheres for proximal vessels, and metal coils for proximal and larger arteries. All abnormal vessels supplying the area of interest were embolized if technically possible. When no underlying pulmonary abnormality was detectable, all bronchial arteries that could be catheterized were embolized. Contraindications to embolization were visualization of the anterior spinal branch or catheter instability. Pulmonary angiography was not routinely performed.

### Statistical analysis

In this paper we present 13 years between 1999–2011 of hospital embolization results in patients with severe haemoptysis. Lineal regression was applied to compute the series’ tendency throughout these 13 years of recorded data.

The daily and monthly distributions of embolizations were used to study the weekly and monthly seasonal indexes [11].

## Results

257 patients who underwent 306 embolizations were included in the analysis.

### Patients

Characteristics of the patients included in the study are described in Table 1. 

**Table 1:** Characteristics of the patients included in the study

	
Age (years)	
Mean (SD)	58.6 (15)
Sex (n, (%))	
Male	190 (74)
Female	67 (26)
Pathological condition [n (%)]	
Bronchiectasis	85 (33)
Cancer	57 (22)
COPD	51 (20)
Active tuberculosis	15 (6)
Idiopathic	49 (19)
Embolizations per patient [n(%)]	
1	221 (86)
2	28 (11)
3	6 (2.2)
4	0 (0)
5	1 (0.4)
6	1 (0.4)

### Temporal tendency

**Figure 1:**
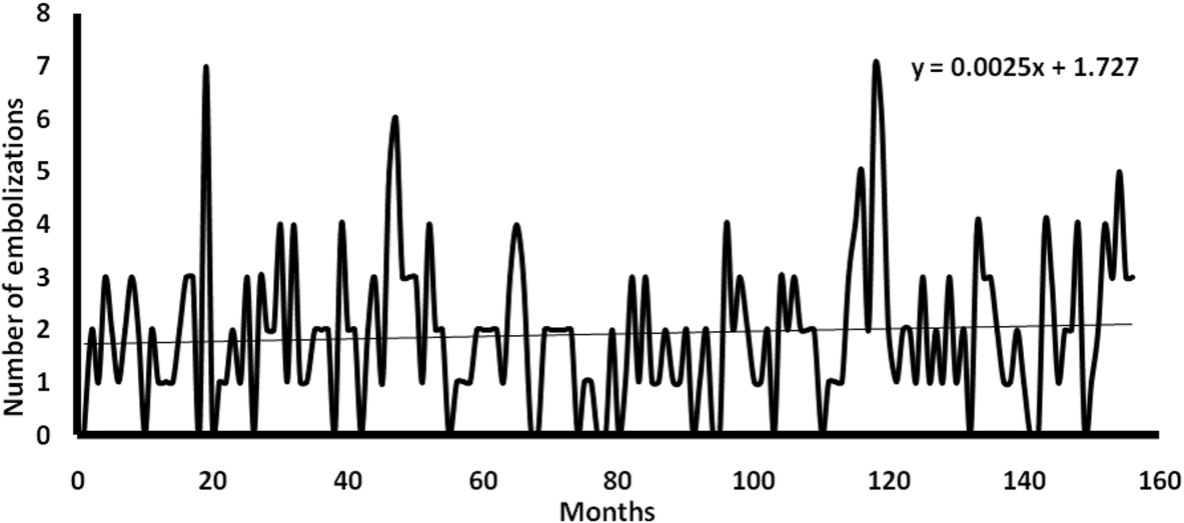
Hemoptysis time series along 156 months between 1999 and 2011.

Monthly accumulative time series during 156 months of recording data is shown in Figure 1. Lineal regression shows a positive tendency with time (y = 0.0025 (time) + 1.727; R^2^ = 0.0063; p < 0.01). 

### Daily variation

**Figure 2:**
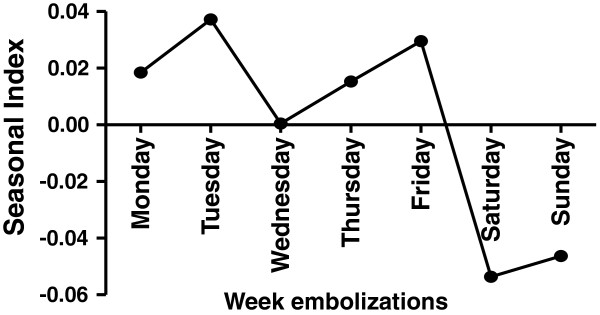
**Seasonal index of weekly embolizations.** There are two peaks on Tuesday and Friday.

Weekday distribution of embolizations for hemoptysis is shown in Figure 2. There are two peaks (Tuesday and Friday), and the figure shows a decrease in the number of BAE during the weekend. 

### Monthly variation

**Figure 3:**
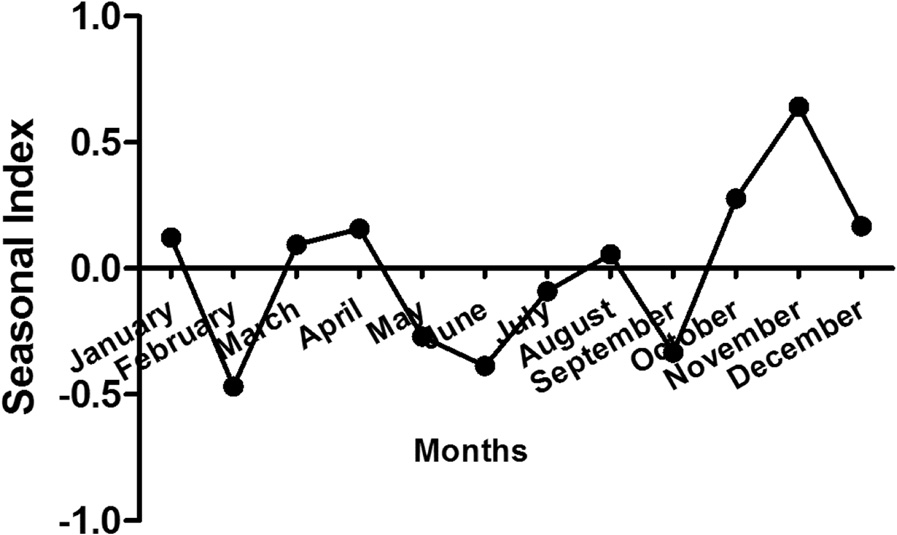
**Seasonal index of the embolization time series.** There are two seasonal peaks in November and April.

Monthly variation in embolizations for hemoptysis is shown in Figure 3. Hemoptysis requiring BAE occurred with some monthly variation demonstrated by two monthly peaks, the first one occurring in April and the second one in November. 

**Figure 4:**
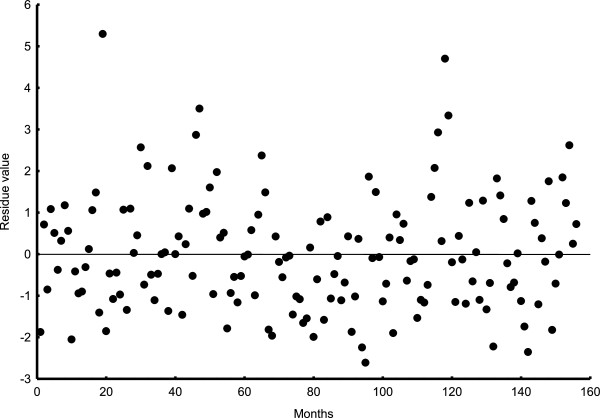
**Residual values of additive seasonal model with time (months).** There is a random distribution of residual values with time. Regression pending was near zero.

Additive model (tendency + month seasonal index) was evaluated by means of residue analysis (model value - real value) (Figure 4). Residual lineal regression with time was not significant (y = −0.003-2.09E^-5^x, p = 0.99). We concluded that the residue series is a random series. 

## Discussion

Several findings emerge from this study. Firstly, we demonstrated that the BAE number has increased with time. Secondly, our results show that there was a decrease in the number of embolizations during the weekend, and last, we found that there were two seasonal peaks, occurring in April and November.

The increase in the number of BAE could be due to the increased utilization of interventional radiology [12]. The decrease in the number of embolizations during the weekend could be attributable to the fact that at our institution interventional radiologists on duty are on call at home at night and during the weekend.

In our study, hemoptysis requiring BAE peaked in April and November. This finding differs from the results of other studies. Boulay and colleagues, for example, reported an increase in cryptogenic hemoptysis requiring hospitalization in late winter and early spring (peak in March) [1]. The same authors, in another article, reported that hemoptysis showed aggregates of clusters in winter [2].

One possible explanation for the increase in the number of patients with hemoptysis in winter could be due to the association of hemoptysis with acute infection [3,4]. Smidt [13] reported that patients with cryptogenic hemoptysis often occurred during time periods when influenza or pneumonia were more frequent. Nevertheless, to the best of our knowledge, no study has evaluated how influenza season correlates with hemoptysis.

Apart from infection, environmental triggering factors have been described in the literature as explanation for hemorrhagic events. Principally, a marked increase in hospital attendance due to epistaxis during colder days has been described [14], although Bray and colleagues, in a larger series found no correlation between temperature, seasonal prevalence and epistaxis [15].

Apart from epistaxis, seasonal variations in other hemorrhagic situations have been described [16-19]. In some of these cases, an association of bleeding occurrence with meteorologic variations was found [17-19]. Boulay et al. [16] described seasonality of both mortality and hospitalization due to variceal bleeding, with the first one peaking in winter, and the latter in winter-spring. Tahri et al. [18] found a higher risk of esophageal varices rupture during winter. Furthermore, they also found a significant correlation between the risk of bleeding and mean temperature, rainfall and stormy weather [18]. The occurrence of subarachnoid hemorrhage has also been associated with meteorological variations [17,19]. Lejeune and colleagues [17] found that aneurysmal bleeding was significantly associated with low maximal temperature, and Setzer et al. [19] demonstrated that atmospheric pressure changes of more than 10 hPa within 24 hours were an independent predictor of clustering of patients with subarachnoid hemorrhage.

In respiratory medicine, seasonal pattern of different diseases have been described. Several articles have examined the seasonal pattern of asthma hospitalizations [20-22] and deaths due to asthma [21-23]. Crighton and colleagues found a clear seasonal pattern for asthma hospitalizations, with peaks occurring between September and November and throughout July and August [20]. Fleming et al. found that the seasonal pattern of asthma evolved with age [23]. Other situations in which a seasonal trend has been described are spontaneous pneumothorax [24] or fatal pulmonary embolism [25].

The main limitation of our study is that we used a therapeutic technique as an indirect indicator of hemoptysis. Thus, not all patients with hemoptysis are included in this study. Those who died due to massive hemoptysis prior to BAE, or those with mild or moderate hemoptysis which did not require BAE were not included in the study.

However the main strength of this report is that all patients included in the study had confirmed severe hemoptysis, and other conditions which could be considered as hemoptysis (such as hematemesis) have been excluded. Another strength is its long follow-up period.

## Conclusion

In summary, we have shown that hemoptysis requiring BAE occurred with some monthly variation demonstrated by two monthly peaks, the first one occurring in April and the second one in November. This seasonal trend might be due to the different prevalence of respiratory tract infections or to some climate variables. Confirmatory studies on the pattern of seasonal variation of hemoptysis shown in the present study are desirable, utilizing large data sets with meteorological variables. An identification of significant environmental factors could be useful to improve preventive measures.

## Competing interests

The authors do not have any financial or personal relationships with people or organizations that could inappropriately influence their work in the present article.

## Authors’ contribution

IG-O,JS-S, FA, ES-M, JR-M, conception and design. JS, JM, JMM, bronchial artery embolization. JAF, IG-O, analysis and interpretation. IG-O, JAF, JS-S, FA, ES-M, JS, JM, JMM, JR-M, drafting manuscript. All authors read and approved the final manuscript.
